# The CHANGE (Climate Health ANalysis Grading Evaluation) tool for weight of evidence reviews on climate change and health research

**DOI:** 10.1186/s12940-023-01040-4

**Published:** 2024-01-19

**Authors:** Nadav L. Sprague, Stephen P. Uong, Hannah Zonnevylle, Trinish Chatterjee, Diana Hernández, Andrew G. Rundle, Christine C. Ekenga

**Affiliations:** 1 Department of Epidemiology, Mailman School of Public Health, Columbia University, 722 W 168th St, New York, NY 10032 USA; 2 Soil and Crop Sciences, School of Integrative Plant Science, College of Agriculture and Life Sciences, Cornell University, Ithaca, NY USA; 3 Department of Earth and Environmental Sciences, Columbia University, New York, NY USA; 4 Department of Sociomedical Sciences, Mailman School of Public Health, Columbia University, New York, NY USA; 5 Gangarosa Department of Environmental Health, Rollins School of Public Health, Emory University, Atlanta, GA USA

**Keywords:** Global warming, Systematic review, Quality and bias assessment, Environmental health, Human health, Environmental exposures, meta-analysis, Scoping review, Biomedical, Climate change and health

## Abstract

**Background:**

Climate change has been identified as one of the biggest threats to human health. Despite this claim, there are no standardized tools that assess the rigor of published literature for use in weight of evidence (WOE) reviews. Standardized assessment tools are essential for creating clear and comparable WOE reviews. As such, we developed a standardized tool for evaluating the quality of climate change and health studies focused on evaluating studies that quantify exposure-response relationships and studies that implement and/or evaluate adaptation interventions.

**Methods:**

The authors explored systematic-review methodology to enhance transparency and increase efficiency in summarizing and synthesizing findings from studies on climate change and health research. The authors adapted and extended existing WOE methods to develop the CHANGE (Climate Health ANalysis Grading Evaluation) tool. The resulting assessment tool has been refined through application and subsequent team input.

**Results:**

The CHANGE tool is a two-step standardized tool for systematic review of climate change and health studies of exposure-response relationships and adaptation intervention studies. Step one of the CHANGE tool aims to classify studies included in weight-of-evidence reviews and step two assesses the quality and presence of bias in the climate change and health studies.

**Conclusion:**

The application of the CHANGE tool in WOE reviews of climate change and health will lead to increased comparability, objectivity, and transparency within this research area.

**Supplementary Information:**

The online version contains supplementary material available at 10.1186/s12940-023-01040-4.

## Background

The World Health Organization has identified climate change as one of the biggest threats to human health [1]. Numerous studies have been published reporting on the impacts of climate change on human health across the globe [2–4], as well as climate change’s impact on exacerbating health inequities [3, 5]. The implications of climate change on human health have been investigated by a broad range of fields, including public health, medicine, ecology, biology, the social sciences, and by transdisciplinary teams drawing from these fields [6–10]. In medicine and public health, weight-of-evidence (WOE) reviews are utilized to assess the direction, validity, accuracy, strength of association, precision, and risk of bias in studies on a specific topic [8, 11]. These WOE reviews are traditionally done through scoping reviews, systematic reviews, and meta-analyses [8, 11]. WOE reviews rely on standardized tools to assess the quality of studies included in these WOE reviews [8, 11]. However, to the authors’ knowledge, such a standardized tool does not currently exist to examine studies of the effects of climate change on human health.

The field of climate change and health research is experiencing a remarkable surge in the number of publications [12, 13]. This field is now generating sufficient peer-reviewed research publications to warrant the use of formalized WOE approaches to address the growing need for synthesizing this literature. In fact, there have been many scientific calls to increase the standardization and transparency of synthesized literature on climate change research [14–16]. Conventional environmental health research, and the current tools for assessing the quality of environmental health studies, typically delve into specific pollutants or hazards. However, climate change is not a conventional environmental health exposure; rather climate change is a global crisis and a set of processes that extends beyond the conventional geographic or political boundaries of conventional environmental exposures [17, 18].

Climate change and health research encompasses a broader scope and scale than conventional environmental health research, examining the complex and interconnected global systems influenced by climate change, such as the atmosphere, hydrosphere, and anthroposphere. Traditional environmental health research may focus solely on one of these aspects while ignoring the interplay and mixtures between them all, thus leading to incomplete conclusions. Additionally, the temporal perspective of climate change research stands apart from that of conventional environmental health research in that climate change research often adopts a longer-term perspective, scrutinizing health impacts that can unfold over decades or even centuries such as the health impacts of sea-level rise and shifting disease patterns. The interconnected and global scope of climate change and health research as well as its long-term perspective can introduce potential threats to study validity and generalizability, particularly when studies focus solely on one exposure, region, or employ too short a time frame. Further, these interconnected climate change exposures give rise to various health outcomes across different typologies including: mortality, direct and indirect physical health impacts, direct and indirect mental health impacts, upstream health determinants, and more [19]. Hence, a tool to assess the quality of climate change and health studies should gather data on the specific type of climate change exposure under study, in addition to health outcome types, geographic scale, and timeframe of analyses.

Due to the complex nature and wide-ranging impacts of climate change, transdisciplinary and comprehensive methods to climate change research, mitigation, and adaptation are a necessity [20]. While conventional environmental health research tends to operate within the confines of a single discipline, climate change and health research necessitates collaboration among experts in climate science, ecology, public health, epidemiology, the social sciences, and other fields to understand its multifaceted impacts on health. Consequently, this diversity in expertise gives rise to a variety of differing theoretical and conceptual approaches to studying the complex interactions between climate change and health. To address this, a study quality assessment tool must record the theoretical and conceptual approaches employed in each study, enabling the evaluation the differing approaches. Additionally, the issue of climate change is politically charged and conflicts of interest may arise as economic actors with stakes in such research are funding climate and health research [21–23]. However, prior environmental health WOE tools do not include questions on funding mechanisms of the studies and the affiliation of the studies’ authors. Prior tools also neglect to incorporate if the study engages with indigenous or community knowledge. Incorporating indigenous knowledge and insights from impacted communities is important in health research, and even more crucial when addressing climate change and health [24–28]. The incorporation of indigenous and community knowledge is essential for ensuring that proposed mitigation and adaptation strategies are culturally relevant [29, 30].

A prior WOE tool, developed by scientists from the US National Institute of Environmental Health Sciences’ Office of Health Assessment and Translation (OHAT), describes a flexible seven-step review process that can be amenable for climate change and health questions [11]; however, the quality assessment step (step 5) is not sufficient for the diversity of environmental exposures associated with climate change-related events and does not account for any other metrics for scientific rigor or quality (such as study classifications, transparency, and covariate selection). In response to this gap and drawing from the previously mentioned shortcomings of pre-existing tools used in related fields and topics, the authors developed the standardized CHANGE (Climate Health Analysis Grading Evaluation) tool for evaluating studies that quantify links between exposure to climate change related variables and health outcomes and for studies that implement and/or evaluate adaptation interventions. The CHANGE tool is a tool to assess the quality of research studies included in WOE reviews that can be seamlessly integrated into step 5 of Rooney et al.‘s systematic review framework [11]. The CHANGE tool incorporates essential WOE metrics, while also addressing the unique aspects of climate change and dimensions that were previously overlooked in prior environmental health WOE assessments.

## Methods

We explored prevailing systematic-review methodologies to enhance transparency and increase efficiency in summarizing and synthesizing findings from studies on climate change and health research. In developing the CHANGE tool, we adapted and extended existing methods from clinical medicine, environmental health, epidemiology, earth science, sociology, biology, and climate change science [8, 11, 14, 31–38]. First, the lead author (N.L.S.) conducted a scoping review of systematic review tools in the aforementioned fields and then compiled the questions included in the tool. Then, the lead author and two additional authors (S.P.U. & C.C.E.) reviewed the list of questions and developed the first iteration of the tool. The first iteration of the tool was applied to a systematic review focused on evaluating the impacts of climate change on mental health in Western Africa, the results of which will be published elsewhere. After conducting the systematic review, the three authors refined the tool based on the insight gained from the initial review. Then, the three authors assembled a team of transdisciplinary experts to further refine and ultimately develop the resulting CHANGE tool. In light of this paper’s focus on climate and health research, particularly in quantifying exposure-response relationships and assessing adaptation interventions, the transdisciplinary team comprised a diverse range of experts, including experts in epidemiology, environmental health sciences, ocean and climate physics, ecology, soil and crop sciences, and sociology.

## Results

We present a two-step standardized CHANGE tool to assess the quality of climate change and health studies (Additional file 1). This two-step standardized tool was adapted from previous existing methods and tools in related fields [8, 11, 14, 31–38]. Because climate and health scientific literature is diverse and transdisciplinary, the authors agreed that the first step of the CHANGE tool must be used to categorize the study being reviewed. The second step of the CHANGE tool will then be used to assess the quality and presence of bias in the study.

### CHANGE Tool Step 1: Study classification

Step one of the CHANGE tool is presented in Table 1. This step aims to classify studies for WOE reviews. The classification step allows researchers to categorize the diverse literature base of climate change and health research. Through this categorization, the systematic review team is empowered to develop more detailed and precise conclusions in the current evidence base, which in turn will help with dissemination of scientific information. In this section, the systematic review team should mark all answers that apply to each question.

**Table 1:** CHANGE Tool Step 1: Study classification

Study Classification Metric	Options
Exposure type	Precipitation; Temperature; Humidity; Drought; Air Pollution; Sea level rise; Storm surge; Flooding; Wildfires; Extreme heat/heat wave; Climate variation; Power outages; Food insecurity/famine; Resource availability; Economic/Market conditions; Climate change adaptation/mitigation (e.g. greening or cooling centers); General; Other
Outcome type	Mortality; Direct physical health impact; Indirect physical health impact; Direct mental health impact; Indirect mental health impact; health systems capacity; Upstream health determinants; Other
Timeframe of climate change exposure	Long term change; Inter-annual or decadal variability; Isolated extreme events
Timeframe of outcome	Long term change; Longitudinal change; Cross-sectional
Spatial scale of the exposure	Individuals; Households; Community; Regional; Sub-national; National; Continental; Global
Spatial scale of the outcome	Individuals; Households; Community; Regional; Sub-national; National; Continental; Global
Regional focus	Global; North America; Europe; Africa; Asia; Central America/South America/the Caribbean; Oceania; Antarctica
Target population	General population; Infants and toddlers (0–2); Children (3–18); Older adults (65+); Women; Pregnant individuals and fetuses; LGBTQIA+ / sexual and gender minorities; Low income groups / groups of low socioeconomic status; Specific racial groups; Specific ethnic groups; Indigenous people; Incarcerated individuals; Immigrants; Outside workers (e.g. farmers; construction workers; etc.); Differently abled persons; Persons with pre-existing medical conditions (e.g. asthma; diabetes; cancer; etc.) or persons with electronic medical devices; Persons with cognitive impairments; Other
Engaging/Incorporating Indigenous and community knowledge	Indigenous knowledge; community knowledge
Study design methodology type	Quantitative; Qualitative; Mixed-methods
Theoretical/conceptual approach	Clinical intervention; Public health intervention (non-clinical); Epidemiological causal theory; Environmental justice/climate justice; Supply-demand economics; Ecosystem services; Ecology/environmental preservation/ecosystem management; Geologic; Land management/agriculture/forestry; Ocean and coastal management; Disaster risk management; Education and tourism; Social determinants; Cultural preservation; Resource and food security; Other
Publishing access	Open access publishing; Non-open access publishing
Funding type	Fossil-fuel industry funding (directly from a corporation, or via industry associations, or industry-funded/industry-aligned philanthropic groups); Other industry (private) funding (directly from a corporation, or via industry associations, or industry-funded/industry-aligned philanthropic groups); Government funding; Foundation/philanthropic funding (independent of industry stakeholders); Academic institution funding; Research stated to be unfunded; Funding not reported
Author affiliations	No authors report affiliations with stakeholder industries or for-hire consulting firms; One or more authors report affiliations with stakeholder industries or for-hire consulting firms

### CHANGE Tool Step 2: Study quality and bias assessment

Table 2 presents step two of the CHANGE tool. For this step, we have developed a 4-tier rating system for ranking each question to align and surpass the current best practices in environmental health research [11, 32, 36]. The rating system is as follows: “1” indicates highest scientific rigor, “2” indicates strong scientific rigor, “3” indicates weak scientific rigor, and “4” indicates poor or no scientific rigor. For a study to be ranked “1” (highest scientific quality), the systematic review team must believe that the study could not be improved in the specific topic the question covers. If the systematic review team has some issue with the study’s scientific rigor, but overall believe it is strong then it should be graded a “2”. Grade “3” indicates that there are significant issues in the study’s scientific rigor, however, the topic is somewhat addressed. Grade “4” indicates that the study fully disregarded this aspect of scientific quality or bias. For questions that there is no applicable answer, “5” is selected for unknown. For this section of the CHANGE tool, the review team must select only one answer per question per study.

**Table 2:** CHANGE Tool Step 2: Study quality and bias assessment

Category	Question	Rating from 1–4; “1” indicating highest scientific rigor and “4” indicating poor or no scientific rigor. “5” indicates unknown
Transparency	Does the study clearly specify the research question?	
	Does the study clearly state the inclusion and exclusion criteria?	
	Is the research reproducible? Does the study present a full description of study design, including a clear rationale for the spatial scale at which exposure was measured and data availability?	
	Does the study clearly assess the “risk of bias?”	
Selection Bias	Are the individuals selected to participate in the study likely to be representative of the target population?	
Covariate Variable Selection	For non-predictive models: Did the study design or analysis account for the minimally sufficient set of confounding and covariate variables	
	For predictive models: Was the selection of predictor variables clearly described and following best standards?	
Detection Bias	Was the measurement ascertainment of the climate variable well described with detail of the technology, developer, detailed usage, and measurement error of the sensor?	
	Was the measurement ascertainment of the climate variable well described with detail of the frequency and recording of location updates, and the method, period, and duration of data collection clearly specified?	
	Was the climate variable well described with detail of the data availability?	
	Was there a clear justification for the chosen climate exposure and the method of exposure assessment?	
	Can we be confident in the exposure characterization? Confidence requires valid, reliable, and sensitive methods to measure exposure applied consistently across exposure groups.	
	Were confounding variables assessed consistently across groups using valid and reliable measures?	
Selective Reporting Bias	Were all measured outcomes (based on the research aims) reported?	

Step two of the CHANGE tool is divided into 5 subsections: transparency, selection bias, covariate variable selection, detection bias, and selective reporting bias. Each of these subsections consist of one to six questions, with each question scored on a scale from 1 to 4, with “1” indicating highest scientific rigor and “4” indicating poor rigor. We encourage review teams to report mean subsection scores and a mean overall score in their manuscripts. A score of “5” indicates that the answer is unknown or not applicable, and therefore should reported but not calculated in the mean scores. Studies with mean subsection and/or mean overall scores close to 1 indicate high scientific rigor for that specific subsection or for the overall assessment, respectively. Conversely, studies with mean subsection and overall scores close to 4 indicate very low scientific rigor for that subsection or for the overall assessment.

#### Transparency

The first subsection ranks the transparency of each study. Research transparency is a researcher’s ethical obligation to make their data, evidence, analysis, and research design accessible to the public in order to facilitate honest evaluations of their evidenced-based claims [39]. Questions in this subsection include issues such as research question clarity, inclusion and exclusion criteria, research reproducibility, and studies’ bias assessments.

#### Selection bias

This subsection focuses on selection bias issues. We define selection bias as a cause of an observed association between the exposure and outcome in a study population that differs from the association that is present in the target population. Selection bias arises due to a selection process of study population participants that is in some manner associated with both exposure and outcome. Here the study population refers to the individuals included in the study and the target population refers to the individuals to which the study is intended to apply. The questions in this subsection ask questions that evaluate the generalizability and external validity of the study and its findings.

#### Covariate variable selection

The following subsection is concerned with issues regarding covariates selected for analyses as potential confounders, mediators, or predictor variables. For non-predictive models, inappropriate covariate selection may cause confounding bias. Confounding bias occurs when confounding variables are not appropriately accounted for, resulting in a distorted measure of association between the exposure and outcome. We define a confounder as a variable that is associated with both the exposure variable and the outcome variable and is not an intermediate variable on the pathway from exposure to outcome [40]. Graders should select either question 13.a. for non-predictive models or 13.b. for predictive models.

#### Detection bias

The detection bias subsection focuses on issues that may cause distortions in the measurement of associations due to issues in the reliability and validity of measurement exposure or outcome variables. The following questions are aimed to ensure that the measurements of variables are clearly described and consistent both within and between groups. These questions ensure that the methods used are valid, reliable, and sensitive. Valid results in the study are defined as values that were not the result of chance or bias, but rather reflects an objective truth. Examples of a study ensuring validity include using valid study measures and including multiple measures per variable. Reliable and valid methods are those that have instruments that produce consistent results and allows a study to examine the effects of unmeasured confounder on study results.

#### Selective reporting bias

The final subsection is concerned with selective reporting bias. Selective reporting bias occurs when the results of the study are not fully or accurately reported (such as null results). As such, this section is concerned with whether or not all measured outcomes that were discussed in the aims of the paper were reported fully in the study.

## Discussion

Climate change is a global crisis with far-reaching implications transcending national borders. In contrast to conventional environmental health research, climate change health research often takes on multiple roles, not only elucidating the health risks posed by climate change but also exploring strategies for adaptation and mitigation, as well as advocating for policies and practices to curb greenhouse gas emissions. As such, we developed the two-step CHANGE tool to evaluate the quality of studies focused on climate change and human health, following the lead of pre-existing tools in related fields and topics [8, 11, 14, 31–38]. In the two-step CHANGE tool, the first step is targeted to classify the type of climate and health study based on differing categorizations. The first step addresses several gaps of previous WOE tools by encouraging reviewers to 1) define the type of climate change-related exposure, 2) identify the timeframe for such exposure, 3) examine the spatial and regional scale of both climate change-related exposures and outcomes, and 4) consider equity issues based on studies’ target populations and regional focus. Additionally, given the transdisciplinary nature of climate and health research, we recommend identifying the underlining theoretical and/or conceptual approach to the work. We also recommend taking publishing access into account, considering funding sources, and evaluating author affiliations. This first step categorizes climate and health research, allowing for enabling step two to assess the overall study quality and evaluate bias in climate change and health studies on a broader scale, as well as within the specific categories determined in the initial step. Researchers and decision makers can use the synthesized results obtained from this tool to ensure that evidence-based insights inform policy decisions and public health practices effectively.

To ensure the rigor of the systematic review, we encourage review teams to have a minimum of two graders evaluate every study [41]. We suggest that all disagreements in study are discussed between the two graders and a third, in which a consensus can be arrived at between all three graders. Further, we encourage for this tool to be used as step 5 within the flexible seven-step OHAT framework [11].

Recent advances in artificial intelligence (AI) tools, particularly large language models, will change how WOE reviews are conducted [42, 43]. However, there will still be a need to assess the quality of studies included in WOE reviews, a process that might evolve as a collaboration between AI and human researchers or as a process conducted solely by AI. Therefore, tools, such as the CHANGE tool, will be necessary to prompt and organize quality assessments for WOE reviews. Perhaps future systematic reviews will incorporate an “AI CHANGE” tool to assess the quality of published research.

## Conclusion

The use of the CHANGE tool in WOE reviews of climate change and health has the potential to increase comparability, objectivity, and transparency in this research area. Employing this method will enhance communication and clarity on the scientific evidence of climate change and its health impacts through standardized and consistent evaluation of the studies. By summarizing and evaluating the current evidence base in a clear and consistent manner, the CHANGE tool will facilitate the provision of essential and accessible information to policy makers, stakeholders, and the public. Consequently, utilizing the CHANGE tool may lead to better and more effective communication of scientific findings to diverse audiences.

### Supplementary Information

**Additional file 1.** The CHANGE (Climate Health ANalysis Grading Evaluation) Tool.

## Data Availability

N/A.

## Abbreviations

CHANGE: Climate Health ANalysis Grading Evaluation
WOE: Weight of evidence
