# Supplementary material for: The CHANGE (Climate Health ANalysis Grading Evaluation) tool for weight of evidence reviews on climate change and health research
Source: Environ Health. 2024 Jan 19;23:7. doi: 10.1186/s12940-023-01040-4 (PMC10797793; doi:10.1186/s12940-023-01040-4)
Supplement: Supplementary file 1 — Additional file 1. The CHANGE (Climate Health ANalysis Grading Evaluation) Tool. [file 12940_2023_1040_MOESM1_ESM.docx]

**Additional file 1. The CHANGE (Climate Health ANalysis Grading Evaluation) Tool**

**Step 1: Study Classification:** This section is aimed to classify the type of climate and health research. For every study, please mark all applicable answers for each question.

1. Exposure type
   - Precipitation
   - Temperature (Dry Bulb or Unspecified)
   - Humidity / Wet Bulb Temperature
   - Drought
   - Air Pollution
     - List all pollutants that apply: ________________
     - List all pollution sources that apply: ________________
   - Sea level rise
   - Storm surge
   - Flooding
   - Wildfires
   - Extreme heat/heat wave
   - Climate variation
   - Power outages
   - Food insecurity / famine
   - Resource availability
   - Economic/Market conditions
   - Climate change adaptation/mitigation (e.g., greening or cooling centers)
   - General
   - Other*
     - If other, specify _______
2. Outcome type
   - Mortality
   - Direct physical health impact (e.g., morbidity, malnutrition, developmental outcomes, etc.)
   - Indirect physical health impact
   - Direct mental health impact
   - Indirect mental health impact
   - Health systems capacity
   - Upstream health determinants
     - Social determinants
     - War /conflict
     - Climate refugees / evacuations and long-term displacement
     - Property loss or damage
     - Disruption of routines and social networks
     - Economic impacts
   - Other*
     - If other, specify _______
3. Timeframe of climate change exposure
   - Long term change (decades to centuries)
   - Inter-annual or decadal variability
   - Isolated extreme events
4. Timeframe of outcome
   - Long term change (decades to centuries)
   - Longitudinal change
   - Cross-sectional
5. Spatial scale of the exposure
   - Individuals
   - Households
   - Community (neighborhood, community district, census tract)
   - Regional (city or county)
   - Sub-national (province, state, etc.)
   - National
   - Continental
   - Global
6. Spatial scale of the outcome
   - Individuals
   - Households
   - Community (neighborhood, community district, census tract)
   - Regional (city or county)
   - Sub-national (province, state, etc.)
   - National
   - Continental
   - Global
7. Regional focus (skip if a region-specific systematic review)
   - Global
   - North America
     - Canada,
     - USA
     - Mexico
   - Europe
     - Eastern Europe (Belarus, Bulgaria, Czech Republic, Hungary, Poland, Republic of Moldova, Romania, Russian Federation, Slovakia, Ukraine)
     - Northern Europe (Åland Islands, Channel Islands, Denmark, Estonia, Faeroe Islands, Finland, Guernsey, Iceland, Ireland, Isle of Man, Jersey, Latvia, Lithuania, Norway, Svalbard and Jan Mayen Islands, Sweden, United Kingdom of Great Britain and Northern Island)
     - Southern Europe (Albania, Andorra, Bosnia and Herzegovina, Croatia, Gibraltar, Greece, Holy See, Italy, Malta, Montenegro, Portugal, San Marino, Serbia, Slovenia, Spain, The former Yugoslav Republic of Macedonia)
     - Western Europe (Austria, Belgium, France, Germany, Liechtenstein, Luxembourg, Monaco, Netherlands, Switzerland)
   - Africa
     - Eastern Africa (Burundi, Comoros, Djibouti, Eritrea, Ethiopia, Kenya, Madagascar, Malawi, Mauritius, Mayotte, Mozambique, Réunion, Rwanda, Seychelles, Somalia, Uganda, United Republic of Tanzania, Zambia, Zimbabwe)
     - Middle Africa (Angola, Cameroon, Central African Republic of the Congo, Equatorial Guinea, Gabon, Sao Tome and Principe)
     - Northern Africa (Algeria, Egypt, Libyan Arab Jamahiriya, Morocco, Sudan, South Sudan, Tunisia, Western Sahara)
     - Southern Africa (Botswana, Lesotho, Namibia, South Africa, Swaziland)
     - Western Africa (Benin, Burkina Faso, Cape Verde, Cote d’Ivoire, Gambia, Ghana, Guinea-Bissau, Liberia, Mali, Mauritania, Niger, Nigeria, Saint Helena, Senegal, Sierra Leone, Togo)
   - Asia
     - Central Asia (Kazakhstan, Kyrgyzstan, Tajikistan, Turkmenistan, Uzbekistan)
     - Eastern Asia (China, Hong Kong Special Administrative Region of China, Democratic People’s, Republic of Korea, Japan, Mongolia, Republic of Korea)
     - Southern Asia (Afghanistan, Bangladesh, Bhutan, India, Iran (Islamic Republic of), Maldives, Nepal, Pakistan, Sri Lanka)
     - Southeastern Asia (Brunei Darussalam, Cambodia, Indonesia, Lao People’s Democratic Republic, Malaysia, Myanmar, Philippines, Singapore, Thailand, Timor-Leste, Vietnam)
     - Western Asia (Armenia, Azerbaijan, Bahrain, Cyprus, Georgia, Iraq, Israel, Jordan, Kuwait, Lebanon, Occupied Palestinian Territory, Oman, Qatar, Saudi Arabia, Syrian Arab Republic, Turkey, United Arab Emirates, Yemen)
   - Central America, South America and the Caribbean
     - Central America (Belize, Costa Rica, El Salvador, Guatemala, Honduras, Nicaragua, Panama)
     - Caribbean (Anguilla, Antigua and Barbuda, Aruba, Bahamas, Barbados, British Virgin Islands, Cayman Islands, Cuba, Dominica, Dominican Republic, Grenada, Guadeloupe, Haiti, Jamaica, Martinique, Montserrat, Netherlands Antilles, Puerto Rico, Saint-Barthélemy, Saint Kitts and Nevis, Saint Lucia, Saint Martin (French part), Saint Vincent and the Grenadines, Trinidad and Tobago, Turks and Caicos Islands, United States Virgin Islands)
     - South America (Argentina, Bolivia (Plurinational State of), Brazil, Chile, Columbia, Ecuador, Falkland Islands (Malvinas), French Guiana, Guyana, Paraguay, Peru, Suriname, Uruguay, Venezuela (Bolivian Republic of))
   - Oceania
     - (Australia, New Zealand, Norfolk Island, Melanesia (Fiji, New Caledonia, Papua New Guinea, Solomon Islands, Vanuatu)
     - Micronesia (Guam, Kiribati, Marshall Islands, Micronesia (Federated States of), Nauru, Northern Mariana Islands, Palau)
     - Polynesia (American Samoa, Cook Islands, French Polynesia, Niue, Pitcairn, Samoa, Tokelau, Tonga, Tuvalu, Wallis and Futuna Islands)
   - Antarctica
8. Target populations
   - General population
   - Infants and toddlers (0-2)
   - Children (3-18)
   - Older adults (65+)
   - Women
   - Pregnant individuals and fetuses
   - LGBTQIA+ / sexual and gender minorities
   - Low income groups / groups of low socioeconomic status
   - Specific racial groups
     - Please specify target racial group: _____
   - Specific ethnic groups
     - Please specify target ethnic group: _____
   - Indigenous people
   - Incarcerated individuals
   - Immigrants
   - Outside workers (e.g., farmers, construction workers, etc.)
   - Differently-abled persons
   - Persons with pre-existing medical conditions (e.g., asthma, diabetes, cancer, etc.) or persons with electronic medical devices
   - Cognitive impairments
   - Other*
     - If other, specify _______
9. Study engages/incorporates:
   - Indigenous knowledge
   - Community knowledge
   - Nether
10. Study design methodology type
    - Quantitative
      - Randomized control trial
      - Quasi-experimental
      - Case-control
      - Cohort Study
      - Cross-sectional study
      - Case Reports and series
    - Qualitative
      - Phenomenological study
      - Ethnographic study
      - Grounded Theory study
      - Historical study
      - Case study
      - Action research study
    - Mixed-methods
      - Convergent parallel (quantitative and qualitative data collected at the same time, but analyzed separately)
      - Embedded (quantitative and qualitative data collected at the same time, within a larger study design)
      - Quant-to-Qual sequential explanatory design (quantitative collected and analyzed, informing the following qualitative data collection and analysis)
      - Qual-to-Quant sequential explanatory design (qualitative collected and analyzed, informing the following quantitative data collection and analysis)
11. Theoretical/conceptual approach
    - Clinical intervention
    - Public health intervention (non-clinical)
    - Epidemiological causal theory
    - Environmental justice / climate justice
    - Supply-demand economics
    - Ecosystem services
    - Ecology/environmental preservation/ecosystem management
    - Geologic
    - Land management/agriculture/forestry
    - Ocean and coastal management
    - Disaster risk management
    - Education and tourism
    - Social determinants
    - Cultural preservation
    - Resource and food security
    - Other
      - Please specify: _____
12. Publishing Access
    - Open access publishing
    - Non-open access publishing
13. Funding Type:
    - Fossil-fuel industry funding (directly from a corporation, or via industry associations, or industry-funded/industry-aligned philanthropic groups)
    - Other industry (private) funding (directly from a corporation, or via industry associations, or industry-funded/industry-aligned philanthropic groups)
    - Government funding
    - Foundation/philanthropic funding (independent of industry stakeholders)
    - Academic institution funding
    - Research stated to be unfunded
    - Funding not reported
14. Author Affiliations:
    - No authors report affiliations with stakeholder industries or for-hire consulting firms.
    - One or more authors report affiliations with stakeholder industries or for-hire consulting firms.

**Step 2: Scientific rigor:** Please select one answer for each question. A rating of 1 indicates highest scientific rigor, 2 indicates strong scientific rigor, 3 indicates weak scientific rigor, and 4 indicates poor or no scientific rigor. For questions that there is no applicable answer, select 5 for unknown.

Transparency

1. Does the study clearly specify the research question?
2. Yes, this study clearly states a specific research question that is being answered in the paper
3. Yes, this study clearly states a specific research question however the paper does not directly answer the research question
4. The research question is stated vaguely or not clear
5. The research question is not stated or is not answered at all by the paper
6. Unknown
7. Does the study clearly state the inclusion and exclusion criteria?
8. Yes, clearly states ALL inclusion/exclusion criteria
9. Almost all inclusion/exclusion criteria are stated
10. Limited inclusion/exclusion criteria are stated
11. No, inclusion/exclusion criteria are not stated
12. Unknown
13. Is the research reproducible? Does the study present a full description of study design, including a clear rationale for the spatial scale at which exposure was measured and data availability?
14. Yes, methods are clear so that research is reproducible, and data is available by request/publicly available
15. Yes, methods are clear so that research is reproducible, but data is not available among request/publicly available
16. Somewhat reproducible, methods are somewhat clear
17. Not reproducible
18. Unknown
19. Does the study clearly assess the “risk of bias?”
20. Study discusses risk of bias in detail
21. Study somewhat discusses risk of bias
22. Study mentions risks of bias
23. Study does not discuss any risks to bias
24. Unknown

Selection bias

1. Are the individuals selected to participate in the study likely to be representative of the target population?
   1. Highly likely to be representative
   2. Likely to be representative
   3. Somewhat likely to be representative
   4. Unlikely to be representative
   5. Unknown

Covariate variable selection

1. **a. For non-predictive models:** Did the study design or analysis account for the minimally sufficient set of confounding and covariate variables
   1. YES, the minimally sufficient set of confounders were accounted for, as demonstrated by a DAG (directed acyclic graph) or conceptual model
   2. SOMEWHAT: Based on a DAG or conceptual model, the confounders selected were either not fully sufficient or not the minimally sufficient set
   3. SOME confounders were accounted for, however this was NOT based off a DAG or conceptual model
   4. NO, they did not account for confounding or moderating variables
   5. Unknown
2. **b. For predictive models:** Was the selection of predictor variables clearly described and following best standards? Best standards for predictor variables include (1) the predictor variables and the outcome evaluated in a transparent and rigorous fashion, (2) predictor variables and outcome evaluate in the whole sample selected initially, (3) the predictive model was derived including an appropriate spectrum of participants, and (4) the predictive model being validated in a different population predictive model clearly defined with.
   1. Yes, selection of predictor variables was clearly described, following best standards
   2. Mostly: selection of predictor variables was described, but did not follow all of the best standards
   3. Somewhat: selection of predictor variables was described, but did not follow any of the best standards/practices
   4. No: the selection of predictor variables was not described
   5. Unknown

Detection bias

1. Was the measurement ascertainment of the climate variable well described with detail of the technology, developer, detailed usage, and measurement error of the sensor?
   1. Derivation of climate measure was very well described
   2. Derivation of climate measure was well described
   3. Derivation of climate measure was not well described
   4. Derivation of climate measure was not at all described
   5. Unknown
2. Was the measurement ascertainment of the climate variable well described with detail of the frequency and recording of location updates, and the method, period, and duration of data collection clearly specified?
   1. Derivation of climate measure was very well described
   2. Derivation of climate measure was well described
   3. Derivation of climate measure was not well described
   4. Derivation of climate measure was not at all described
   5. Unknown
3. Was the climate variable well described with detail of the data availability?
   1. Yes, the study included public access data
   2. Yes, the data was available among requests
   3. Yes, but the data was not accessible to the public
   4. No, there was no data availability statement
   5. Unknown
4. Was there a clear justification for the chosen climate exposure and the method of exposure assessment?
   1. Justification of climate measure was very well described
   2. Justification of climate exposure was well described, but specific measure was not well described
   3. Justification of climate exposure was not well described, but specific measure was well described
   4. Justification of climate measure was not at all described
   5. Unknown
5. Can we be confident in the exposure characterization? Confidence requires valid, reliable, and sensitive methods to measure exposure applied consistently across exposure groups.
6. Yes
7. No–the methods WERE NOT valid, reliable, and/or sensitive, but WERE applied consistency across groups
8. No–the methods WERE valid, reliable, and/or sensitive, but WERE NOT applied consistency across groups
9. No–the methods WERE NOT valid, reliable, and/or sensitive AND WERE NOT applied consistency across groups
10. Unknown
11. Were confounding variables assessed consistently across groups using valid and reliable measures?
12. Yes
13. Confounding variables were assessed. However, this was either NOT consistent -OR- NOT using fully valid and reliable measures
14. Confounding variables were assessed. However, this was NOT consistent AND NOT using fully valid and reliable measures
15. Confounding variables were NOT assessed
16. Unknown

Selective Reporting Bias

| 1. Were all measured outcomes (based on the research aims) reported? |
| --- |

1. Yes, all measured outcomes were reported and appropriately analyzed
2. Yes, all measured outcomes were reported, but not all were analyzed
3. Not all measured outcomes were reported, but a rationale was provided onto why they were not included
4. No
5. Unknown

1. Rooney AA, Boyles AL, Wolfe MS, Bucher JR, Thayer KA. Systematic Review and Evidence Integration for Literature-Based Environmental Health Science Assessments. *Environmental Health Perspectives*. 2014;122(7):711-718. doi:doi:10.1289/ehp.1307972

2. Sprague NL, Bancalari P, Karim W, Siddiq S. Growing up green: a systematic review of the influence of greenspace on youth development and health outcomes. *Journal of Exposure Science & Environmental Epidemiology*. 2022:1-22.

3. Eick SM, Goin DE, Chartres N, Lam J, Woodruff TJ. Assessing risk of bias in human environmental epidemiology studies using three tools: different conclusions from different tools. *Systematic reviews*. 2020;9(1):1-13.

4. Higgins J, Altman D, Sterne J. Chapter 8: Assessing risk of bias in included studies. Cochrane Handbook for Systematic Reviews of Interventions Version 5.1. 0 [updated March 2011]. *The Cochrane Collaboration*. 2011:187-214.

5. Woodruff TJ, Sutton P. The Navigation Guide Systematic Review Methodology: A Rigorous and Transparent Method for Translating Environmental Health Science into Better Health Outcomes. *Environmental Health Perspectives*. 2014;122(10):1007-1014. doi:doi:10.1289/ehp.1307175

6. Lachowycz K, Jones AP. Greenspace and obesity: a systematic review of the evidence. *Obes Rev*. May 2011;12(5):e183-9. doi:10.1111/j.1467-789X.2010.00827.x

7. Weilnhammer V, Schmid J, Mittermeier I, et al. Extreme weather events in europe and their health consequences–A systematic review. *International Journal of Hygiene and Environmental Health*. 2021;233:113688.

8. Berrang-Ford L, Ford JD, Paterson J. Are we adapting to climate change? *Global Environmental Change*. 2011/02/01/ 2011;21(1):25-33. doi:<https://doi.org/10.1016/j.gloenvcha.2010.09.012>

9. Berrang-Ford L, Pearce T, Ford JD. Systematic review approaches for climate change adaptation research. *Regional Environmental Change*. 2015/06/01 2015;15(5):755-769. doi:10.1007/s10113-014-0708-7

10. Jia P, Yu C, Remais JV, et al. Spatial Lifecourse Epidemiology Reporting Standards (ISLE-ReSt) statement. *Health & Place*. 2020/01/01/ 2020;61:102243. doi:<https://doi.org/10.1016/j.healthplace.2019.102243>

11. Luo G, Nkoy FL, Stone BL, Schmick D, Johnson MD. A systematic review of predictive models for asthma development in children. *BMC Medical Informatics and Decision Making*. 2015/11/28 2015;15(1):99. doi:10.1186/s12911-015-0224-9
